# DFT and molecular docking investigations of oxicam derivatives

**DOI:** 10.1016/j.heliyon.2019.e02175

**Published:** 2019-07-30

**Authors:** Y.Shyma Mary, Y.Sheena Mary, K.S. Resmi, Renjith Thomas

**Affiliations:** a Department of Physics, Fatima Mata National College (Autonomous), Kollam, Kerala, India; b Department of Chemistry, St. Berchmans College (Autonomous), Changanassery, Kerala, India

**Keywords:** Organic chemistry, Theoretical chemistry, Pharmaceutical chemistry, DFT, MEP, FT-IR, FT-Raman, Molecular docking

## Abstract

The organic molecule tenoxicam and similar derivatives, piroxicam and isoxicam have been studied by quantum chemical theory (DFT), FT-Raman and FT-IR. By FMOs energies the charge transfer inside the molecules are obtained. The UV-Vis spectra of the compounds are simulated to study the electronic transition in the target molecules. By using natural bond orbital (NBO), charge delocalization analyzes arising from hyper conjugative interactions and the stability of the molecules are obtained. First order hyperpolarizability of piroxicam is higher than that of isoxicam and tenoxicam. The reactive areas are thoroughly studied by MEP. Prediction of Activity Spectra gives activities, anti-inflammatory, CYP2C9 substrate and gout treatment. Docked ligands form a stable complex with the receptors.

## 1 Introduction

Oxicams are enolcarboxamides that exhibit number of pharmacological properties and effective for postoperative pain, arthritis, degenerative joint diseases and osteoarthritis [1]. Tenoxicam is a nonsteroidal anti-inflammatory drug that is a part of the oxicam family and it can be used as an effective analgesic and antipyretic agent [2]. Piroxicam possess multifunctional activity including chemoprevention and its photochemical properties are sensitive to medium [3, 4]. Tamasi et al. [5] reported the synthesis and DFT studies of oxicam complexes. By giving the mol files of tenoxicam, piroxicam and isoxicam in the software it predicts different biological activities. Literature survey shows that there is no detailed study done on the molecules both quantum chemical and experimental spectroscopic studies which are very essential for micro level function of any organic compounds. The structural and physio-chemical properties of the compounds can be found out by spectroscopic and quantum computational tools like Density Functional Theory. These structural and physio-chemical properties can be used to establish relationships between these properties and biological activity of the compound [6]. Due to a large number of applications of NLO materials in optoelectronic technology, the molecules have been analyzed for their hyperpolarizability [7]. Several properties like highest occupied molecular orbital, lowest unoccupied molecular orbital energies, various chemical descriptors, molecular electrostatic potential analysis are carried out to provide information about charge transfer within the molecules. The spectral analysis of tenoxicam, piroxicam and isoxicam are performed and compared with theoretical values. The redistribution of electron density are investigated.

## 2 Calculation

**Fig. 1:**
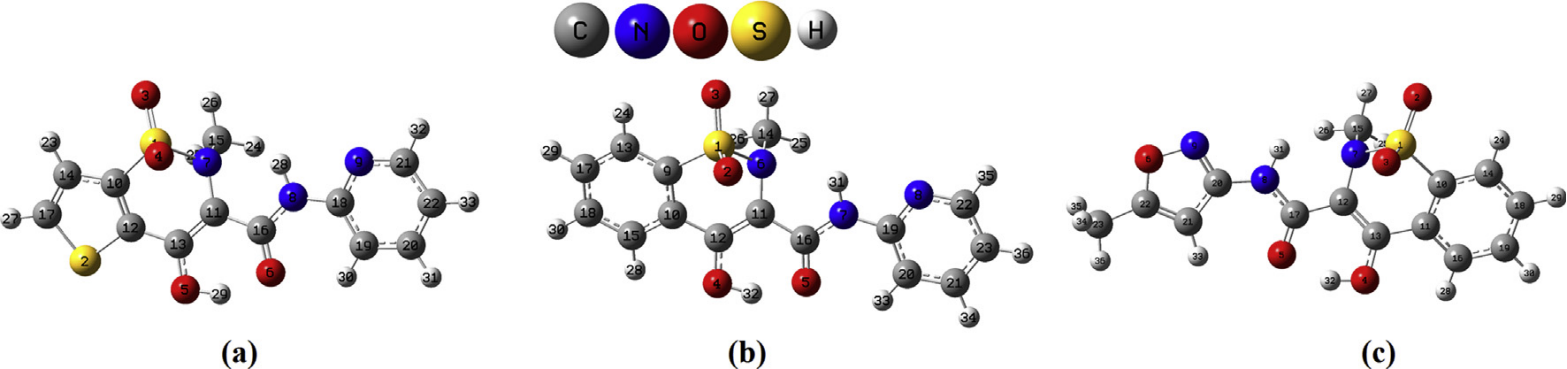
Optimized geometry of (a) tenoxicam, (b) piroxicam and (c) isoxicam.

All calculations are performed using the Gaussian09 software package [8]. DFT method was employed using B3LYP functional and cc-pVDZ (5D, 7F) basis set. Results from frequency calculations after scaling were used to get the IR spectral data, which is compared with the experimental spectral vibrations [9]. By using the TD-DFT method the electronic properties of the molecules (Fig. 1) determined using CAM-B3LYP functional and cc-pVDZ basis set. The spectral data are obtained from Bio-Rad Laboratories, Inc. SpectraBase [10].

## 3 Results and discussions

### 3.1 Natural bond orbital analysis

NBO analysis provides information about various hyper conjugative interactions and intermolecular charge transfer between bonding and antibonding orbitals. In the current work the analysis has been done using DFT method at B3LYP/cc-pVDZ (5D, 7F) level. The stabilization energy forms an important characteristic in this analysis and higher this energy, greater will be the interaction between the electron donors and hence greater the extent of conjugation. Intra molecular interactions are very much important in predicting the stability and reactivity of the target molecules [11]. To study intra and inter-molecular non-bonded interactions the NBO is the efficient method for organic and bio-molecular compounds [12]. Based on the second order perturbation theory the important donor-acceptor interactions are calculated. The important interactions are: For tenoxicam: The strong interactions are N8→π*(O6-C16), N8→π*(C18-C19), O6→ σ*(N8-C16), O5→π*(C11-C13), O4→ σ*(S1-C10), O4→ σ*(S1-O3), O3→ σ*(S1-C10), O3→ σ*(S1-O4), S2→π*(C14-C17), S2→π*(C10-C12), C18-C19→π*(C20-C22), C18-C19→π*(N9-C21), C11-C13→π*(O6-C16) with energies 79.05, 38.52, 21.56, 48.55, 22.48, 16.29,20.19, 17.93, 21.35, 26.13, 22.73, 17.71, 26.08 kcal/mol. For piroxicam: N7→π*(O5-C16), N7→π*(C19-C20), O5→ σ*(N7-C16), O4→π*(C11-C12), O3→ σ*(S1-N6), O3→ σ*(S1-C9), O2→σ*(S1-N6), O2→ σ*(S1-C9), C19-C20→π*(N8-C22), C19-C20→π*(C21-C23), C11-C12→π*(O5-C16) with energies, 79.26, 38.71, 21.48, 48.24, 34.03, 19.38, 33.32, 22.57, 17.71, 22.81, 25.94 kcal/mol and for isoxicam: N8→π*(O5-C17), N8→π*(N9-C20), O6→π*(N9-C20), O6→π*(C21-C22), O5→ σ*(N8-C17), O4→π*(C12-C13), O3→ σ*(S1-N7), O2→ σ*(S1-N7), O2→ σ*(S1-O3), O2→ σ*(S1-C10), C21-C22→π*(N9-C20), C12-C13→π*(O5-C17), C10-C14→π*(C11-C16) with energies, 74.85, 46.86, 16.12, 35.11, 22.01, 48.41, 33.52, 34.25, 18.74, 19.44, 28.36, 26.52, 19.66 kcal/mol. The delocalization energies are very high and hence the molecules are stable enough to show desired medicinal properties.

### 3.2 Electronic spectra and NLO properties

**Fig. 2:**
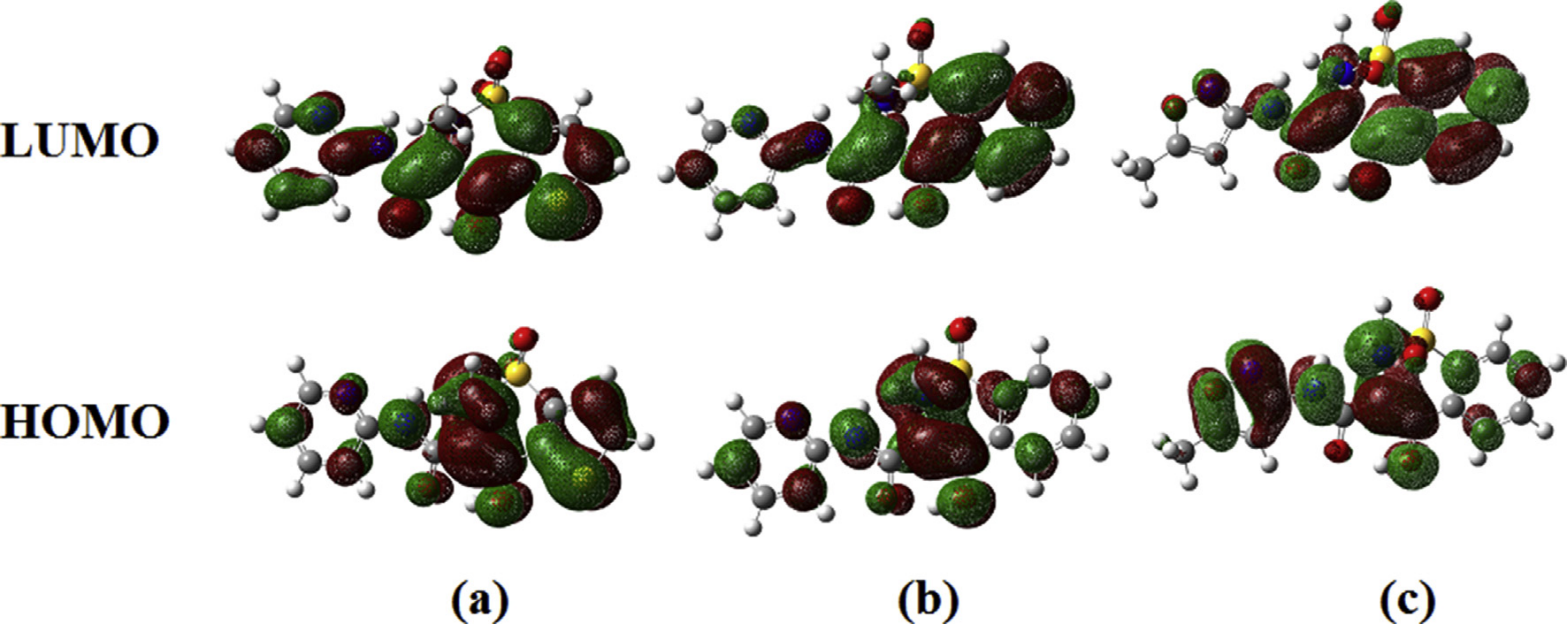
HOMO-LUMO plots of (a) tenoxicam, (b) piroxicam and (c) isoxicam.

The 3D diagrams of HOMO and LUMO are shown in Fig. 2. HOMO represents the donating nature of an electron and LUMO represent accepting nature of electrons [13]. HOMO and LUMO energy are -7.837, -4.931 for tenoxicam, -8.004, -5.315 for piroxicam and -7.867, -5.268 for isoxicam. The band gap energy is 2.908 for tenoxicam, 2.689 for piroxicam and 2.599 for isoxicam explains the ultimate transfer of charge happening within the molecule and shows the biological activity. The values of chemical descriptors are given in Table 1. Due to the low value of HOMO-LUMO energy gap [14] these compounds have high softness nature. The low value of the electrophilicity index suggests the biological activity of the compounds. Nonlinear optical studies are an important part in the present world of researchers as NLO active materials find applications in telecommunication, potential applications in modern communication technology, optical signal processing and data storage [15]. Molecular based nonlinear optical behavior (NLO) materials have current attention and great importance because they involve new technical phenomena owing to the emerging application in electronic devices [16]. First order hyperpolarizability of piroxicam (9.232×10^−30^ esu) > isoxicam (9.112×10^−30^ esu) > tenoxicam (7.756×10^−30^ esu) which are 71, 70 and 60 times that of urea while the second order values are -18.132×10^−37^, -18.336×10^−37^ and -19.060×10^−37^ for tenoxicam, piroxicam and isoxicam [17]. These values show that the title compounds are an important class of compounds in the rank of NLO materials [18]. Electronic transitions in a molecule usually happen in the UV and Visible region of the electromagnetic spectra. Being a time dependent phenomena, original DFT treatment could not explain this phenomenon which involves a change in the electric field of the radiations. For that time dependent density functional theory, known as TDDFT is used to simulate the electronic spectra of the compounds. Long range corrected density functional- CAM-B3LYP is used in this study with the generic 6-31G(d) basis set in methanol solvent cage as provided in the PCM solvation model [19]. In the case of tenoxicam, the DOS spectra show no unusual overlap in the frontier molecular orbitals. Simulated UV spectrum shows two strong excitations at 336.76 nm and 263.69 nm with oscillator strength 0.7602 and 0.103 respectively. The former may be due to the pi to antibonding pi orbital transitions and it is found that HOMO to LUMO transition contributes 75% to it followed by HOMOI-1 to LUMO (21%). The second transition can be attributed to the lone pair to antibonding orbital interactions, hence of low intensity. Data shows that this transition is due to HOMO-LUMO (14%), HOMO-1 to LUMO (60%) and HOMO-3 to LUMO (11%). For the compound pyroxicam, the one dominant transition was at 310 nm with oscillator strength 0.8658 due to the HOMO-1 to LUMO (21%) and HOMO to LUMO (74%), which is due to the pi to pi antibonding transition. There are other two less intense transitions too at 265.48 and 253.00 nm originating from the antibonding orbitals. Isoxicam shows an intense peak at 304.81 nm with intensity 0.6978 and can be attributed to HOMO to LUMO (91%) and HOMO-1 to LUMO (4%). There is another low intense transition originating from the lone pairs at 262.10 nm with oscillator strength of 0.0262.

**Table 1:** Chemical descriptors.

Compound	HOMO	LUMO	I = -EHOMO	A = -ELUMO	Gap	η=(I-A)/2	μ = -(I + A)/2	ω = μ^2^/2η
Tenoxicam	-7.837	-4.931	7.837	4.931	2.908	1.454	-6.384	14.015
Piroxicam	-8.004	-5.315	8.004	5.315	2.689	1.345	-6.660	16.489
Isoxicam	-7.867	-5.268	7.867	5.268	2.599	1.300	-6.568	16.592

### 3.3 Molecular electrostatic potential

**Fig. 3:**
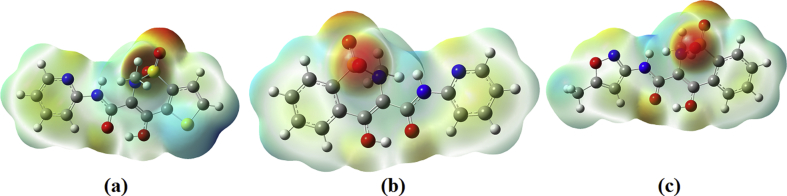
MEP plots of (a) tenoxicam, (b) piroxicam and (c) isoxicam.

MEPs map of the title compounds are shown in Fig. 3 [20]. The various surfaces of the molecule are having different electrostatic potentials and are in different colors. The negative spots are represented by red, blue is the regions of the positive and the green gives zero potential. From the diagram we can see that the negative portions are near the oxygen atoms and the N atom in the ring for all the compounds. The positive areas are around the NH groups. In this molecule, the negative regions attract proton from the amino acids or protein. These active sites are evidence of the biological activity of the title molecules.

### 3.4 IR and Raman spectra

Bands (Table 2) at 3390 (IR), 3411 (DFT) for tenoxicam, 3400 (IR), 3409 (DFT) for piroxicam and 3290 (IR), 3300 (Raman), 3417 (DFT) for isoxicam are assigned as the NH stretching modes [21]. The υC=O is assigned at 1610 (IR), 1605 (Raman), 1614 (DFT) for tenoxicam, 1640 (IR), 1615 (Raman), 1617 (DFT) for piroxicam and 1630 (IR), 1624 (DFT) for isoxicam [21]. The downshift of these NH and C=O modes are due to strong hyper conjugative interactions as given by NBO analysis. The C=C stretching modes are assigned at 1422 (IR), 1500, 1429 (Raman), 1501, 1422 (DFT) for tenoxicam, 1600 (IR), 1600 (Raman), 1598 (DFT) for piroxicam and at 1608, 1600 (IR), 1603 (Raman), 1606, 1590 (DFT) for isoxicam [21]. The SO2 stretching modes are assigned nearly at around 1251 (IR) and 1250 (DFT) for all the three molecules [21]. The CS stretching mode are observed at 785, 653 (tenoxicam), 631 (piroxicam), 632 (isoxicam) in IR, 668, 645 (tenoxicam), 640 (piroxicam and isoxicam) in Raman spectrum [21]. All the experimentally observed bands are identified as assigned.

**Table 2:** Vibrational assignments.

2.1: Tenoxicam					
B3LYP/CC-pVDZ (5D, 7F)			IR	Raman	Assignments^a^
υ(cm^−1^)	IRI	RA	υ(cm^−1^)	υ(cm^−1^)	-
3411	61.99	188.9	3390	-	υNH
3132	4.19	49.90	3145	3140	υCH
3109	3.53	82.61	3105	3110	υCH
3084	16.36	311.8	3088	3085	υCH
2990	13.91	62.94	3000	-	υCH3
2902	31.62	73.61	2915	2920	υCH3
1614	137.2	371.6	1610	1605	υC=O
1566	142.5	303.6	1563	-	υRingI
1501	105.7	1950.4	-	1500	υC=CRingII
1499	368.0	34.43	1495	-	δNH
1422	18.88	562.4	1422	1424	υC=CRingIII
1406	2.99	12.86	-	1404	δCH3
1381	2.03	241.3	-	1380	δOH
1364	6.11	127.2	1370	-	δCH3
1344	152.0	627.7	1345	1347	υRingIII
1315	78.31	80.18	1320	-	δCH
1292	92.89	74.80	1296	1290	υRingI
1253	176.2	5.31	1251	-	υSO2
1247	27.41	126.2	-	1249	υCN
1201	58.53	299.0	1200	1200	υCN
1186	79.13	141.0	-	1188	υCN
1186	79.13	141.0	-	1188	υCN
1141	31.75	18.56	1148	1147	δCH
1130	26.31	47.27	1138	-	δCH3
1102	9.25	72.35	1100	1100	υCC
1082	7.35	5.65	1085	-	δCH3
1046	15.54	48.98	1048	1050	δCH
1009	39.54	3.08	995	1005	υCN
978	0.28	0.56	976	-	γCH
905	83.51	0.71	903	-	γOH
895	14.22	2.80	-	893	δC=O
870	6.93	27.68	-	871	γCH
865	2.01	2.35	858	-	γCH
839	10.64	11.24	840	840	υRingI
781	19.91	4.33	785	-	υCS
769	38.51	0.77	-	770	τRingI
763	16.96	14.04	760	755	υSN
735	40.39	8.93	737	-	τRingI
703	38.57	2.00	703	700	γNH
662	11.20	6.26	-	668	υCS
656	7.09	18.92	653	645	υCS
588	79.05	7.45	590	580	δRingI
531	16.05	3.06	535	536	δSO2
513	35.12	6.20	-	508	δSO2
480	17.16	1.48	478	478	τRingII
455	3.03	1.97	448	452	τRingIII
406	2.37	0.78	403	404	τRingI
305	2.51	11.22	-	303	τRingIII
246	1.65	0.96	-	250	δRingIII
171	2.90	2.94	-	173	τCH3

2.2: Piroxicam					
B3LYP/CC-pVDZ (5D, 7F)			IR	Raman	Assignments^b^
υ(cm^−1^)	IRI	RA	υ(cm^−1^)	υ(cm^−1^)	-
3409	68.10	200.6	3400	-	υNH
3098	2.69	157.6	-	3100	υCHRingIII
3084	16.55	311.8	3084	-	υCHRingI
3062	9.34	120.9	-	3061	υCHRingI
3036	23.86	151.6	3030	3038	υCHRingI
2988	14.68	69.53	2950	2965	υCH3
2900	31.75	72.08	2900	-	υCH3
1617	167.8	141.0	1640	1615	υC=O
1598	76.25	596.6	1600	1600	υC=C
1579	57.84	599.6	1578	1585	υRingI
1566	89.17	119.3	-	1563	υRingI
1545	44.02	131.4	1540	1547	υRingIII
1501	13.91	149.2	-	1498	δNH
1445	23.91	114.6	1447	1443	υRingIII
1409	299.2	2.94	1410	-	υRingI
1406	2.74	31.88	-	1405	δCH3
1364	9.45	112.9	-	1363	δCH3
1344	139.8	620.8	1348	1334	υCO
1305	17.95	24.83	1304	1303	υRingIII
1292	81.25	4.40	1290	-	υRingI
1269	10.86	13.99	-	1272	δCHRingI
1250	176.4	10.55	1250	-	υSO2
1247	7.99	202.3	-	1245	υCN
1211	52.10	222.5	1215	1210	υCN
1185	34.03	74.70	1180	1187	δNH
1138	40.10	88.75	1145	1140	δCHRingIII
1117	6.93	12.80	1120	-	δCHRingI
1101	81.74	132.2	1100	1100	δCHRingIII
1071	26.20	11.67	-	1072	δCHRingI
1065	76.29	7.29	1065	1059	δCHRingII
1028	5.04	29.65	1033	-	δCHRingI
1011	5.14	45.75	-	1008	υRingIII
980	0.53	0.59	985	-	γCHRingIII
978	0.29	0.53	977	-	γCHRingI
943	0.65	1.16	942	942	γCHRingI
873	0.72	4.59	-	875	γCHRingIII
844	11.16	8.81	842	842	δRingI
778	19.45	10.84	-	777	τRingII
751	16.41	10.12	750	750	τRingIII
735	4.03	4.68	738	-	τRingI
703	27.83	3.56	698	702	δRingIII
642	1.31	3.32	-	640	υCS
635	2.26	9.99	631	-	υCS
592	6.43	10.96	-	590	δRingII
552	5.93	7.71	553	550	δRingI
522	35.66	5.43	523	523	τRingII
441	9.47	1.26	445	446	τRingIII
413	4.79	2.93	415	-	τRingIII
406	1.98	0.74	405	-	τRingI
372	2.14	2.23	-	373	τRingII
292	9.25	3.30	-	290	τRingIII
245	1.46	0.90	-	248	τRingI
201	0.18	0.74	-	200	τSO2
167	1.88	2.52	-	170	τCH3

2.3: Isoxicam					
B3LYP/CC-pVDZ (5D, 7F)			IR	Raman	Assignments^c^
υ(cm^−1^)	IRI	RA	υ(cm^−1^)	υ(cm^−1^)	-
3417	75.13	140.7	3290	3300	υNH
3187	6.20	26.83	3180	3180	υCHRingI
3077	8.03	215.5	3075	3078	υCHRingIII
3024	5.34	7.67	3010	-	υCH3
2988	14.04	70.57	-	3000	υCH3
2984	6.43	144.4	-	2984	υCH3
2924	18.09	333.1	2945	2935	υCH3
1624	166.4	4.56	1630	-	υC=O
1606	72.67	48.99	1608	1603	υC=C
1590	405.8	7.93	1600	-	υC=C
1579	2.75	316.0	1580	1570	υRingIII
1546	24.68	33.18	1548	1548	υRingIII
1448	161.2	2.32	1450	-	δNH
1446	44.78	187.9	-	1443	υRingIII
1430	394.5	1.80	1429	-	υC=N
1381	33.06	9.08	1380	-	δOH
1365	6.22	73.39	-	1368	δCH3
1343	21.96	172.3	1347	1347	δCH3
1337	128.3	5.21	1332	-	υCO
1305	28.65	26.13	1298	1300	υRingIII
1251	7.46	24.56	-	1252	υCO
1250	139.5	7.16	1250	-	υSO2
1223	33.45	5.17	1225	-	υCN
1190	14.90	8.44	1200	1188	υCN
1140	40.88	93.06	1145	1145	δCH3
1116	12.25	23.28	1119	1120	δCH3
1067	96.69	7.89	1066	1070	δCHRingIII
1028	7.01	1.96	-	1035	δCHRingI
1016	5.61	6.85	-	1018	δCHRingIII
1011	11.88	5.75	1010	-	υRingIII
1001	7.32	11.65	-	1000	δCH3
983	3.01	14.98	-	985	δCH3
966	0.48	1.93	960	-	δRingI
949	0.73	0.59	946	-	γCHRingIII
908	76.85	0.99	910	910	γOH
894	51.44	2.41	893	892	υNO
813	11.99	6.31	812	816	δRingII
795	21.01	0.87	793	793	γCHRingI
775	15.58	2.02	-	778	γCHRingIII
753	30.39	8.11	752	752	γCHRingIII
740	16.42	4.18	740	-	τRingIII
722	14.50	1.23	-	723	τRingIII
694	55.72	4.92	703	700	δRingI
655	48.79	1.50	653	658	γNH
641	1.35	3.49	-	640	υCS
633	5.02	4.30	632	-	υCS
563	1.49	6.70	558	-	δRingII
545	32.26	1.47	544	-	τRingIII
523	41.26	6.16	515	525	τSO2
441	7.86	1.31	447	440	τRingII
422	4.05	5.81	425	423	τRingIII
397	5.00	8.06	400	400	τRingI
370	11.34	2.96	-	372	δRingII
353	4.66	4.21	-	348	δRingI
306	4.54	3.99	-	304	τRingII
276	5.02	1.30	-	275	τRingIII
244	1.30	3.02	-	247	δRingIII
203	0.08	0.79	-	200	τCH3
177	2.88	2.65	-	175	τCH3

a υ-stretching; δ-in-plane deformation; γ-out-of-plane deformation; τ-torsion;; IR_I_-IR intensity(KM/Mole); R_A_-Raman activity(Ǻ^4^/amu); RingI-pyridine ring; RingII-Ring having SO2; RingIII-Five member ring.

b υ-stretching; δ-in-plane deformation; γ-out-of-plane deformation; τ-torsion;; IR_I_-IR intensity(KM/Mole); R_A_-Raman activity(Ǻ^4^/amu); RingI-pyridine ring; RingII-Ring having SO2; RingIII-Phenyl ring.

c υ-stretching; δ-in-plane deformation; γ-out-of-plane deformation; τ-torsion;; IR_I_-IR intensity(KM/Mole); R_A_-Raman activity(Ǻ^4^/amu); RingI- Five member ring; RingII-Ring having SO2; RingIII-Phenyl ring.

### 3.5 Molecular docking

PASS (Prediction of Activity Spectra) [22] gives (Table 3) activities, anti-inflammatory, CYP2C9 substrate and gout treatment (activity values 0.934, 0.904 and 0.898. Receptors, 3DY9, 4NZ2 and 2AYR were obtained from the protein data bank website. PatchDock Server is used for docking purpose [23, 24, 25, 26].

**Table 3:** PASS prediction for the activity spectrum. Pa represents probability to be active and Pi represents probability to be inactive.

Pa	Pi	Activity
0.934	0.004	Antiinflammatory
0.904	0.004	CYP2C9 substrate
0.898	0.002	Gout treatment
0.862	0.005	Analgesic
0.853	0.005	Antiarthritic
0.794	0.010	CYP2C substrate
0.774	0.004	Non-steroidal antiinflammatory agent
0.759	0.003	Peroxidase substrate
0.729	0.005	Analgesic, non-opioid
0.708	0.004	CYP2C9 inhibitor

For the protein 3DY9: the amino acid interactions are: Amino acid His129 forms H-bond with methylene while Phe13 has π-sulfur interaction with SO_2_ group. Lys132, Lys136 having π-alkyl interaction with pyridine ring and Trp37 shows two π-sulfur interaction with sulphur atom of the thiophene ring for tenoxicam; Lys149 forms H-bond with SO_2_ and Lys150, Ala275 having π-alkyl bond with pyridine ring for piroxicam and Lys149, Gly130, His129 forms H-bond with carbonyl group, SO_2_ group, methyl group respectively while Tys139, Lys136, Lys132 shows π-π-T shaped, alkyl, π-alkyl interaction respectively with the ligand for isoxicam.

For the protein 4NZ2: The residues of Lys421 forms H-bond with methylene and OH while Asp349 shows π-anion interaction with pyridine ring. Lys420, Lys421, Lys423 having π-alkyl interaction with pyridine and phenyl ring where as Phe419 shows π-sulfur interaction with SO_2_ group for tenoxicam; Lys421 forms H-bond with SO_2_ and methyl group as well as π-alkyl interaction with pyridine ring. Lys150, Ala275 having π-alkyl interaction with phenyl ring. His353, Lys420 shows π-π-T shaped, π-alkyl interactions respectively with pyridine whereas Phe419 has a π-sulfur interaction with SO_2_ group for piroxicam and Amino acids Asp414, Asp349 forms π-anion interaction and Arg342 shows π-alkyl interaction with phenyl ring. Lys423, Lys421 shows π-alkyl interaction respectively with the isoxicam**.**

For the protein 2AYR: The residues of amino acid Gly390, Trp393 forms H-bond with C=O and OH while Glu323, Glu353 shows π-anion interaction with thiophene ring and pyridine. Trp393 forms π-sulfur interaction with sulphur atom and Pro324 having π-alkyl interaction with SO_2_ group for tenoxicam; Amino acids Ile326, Gly390, Trp393 forms H-bond with sulphur atom, C=O, OH group respectively while Glu323, Glu353 shows π-anion interaction with pyridine. Pro324, Met357 having π-alkyl interaction with pyridine ring whereas Ile326, Lys35 gives π-sigma, π-alkyl interactions respectively with phenyl ring for piroxicam and Amino acids Glu323 forms H-bond with OH while Arg394 has π-cation interaction with phenyl ring. Leu320, Trp393 having π-alkyl with methyl and Pro324 forms π-alkyl interaction with SO2 group for isoxicam.

**Fig. 4:**
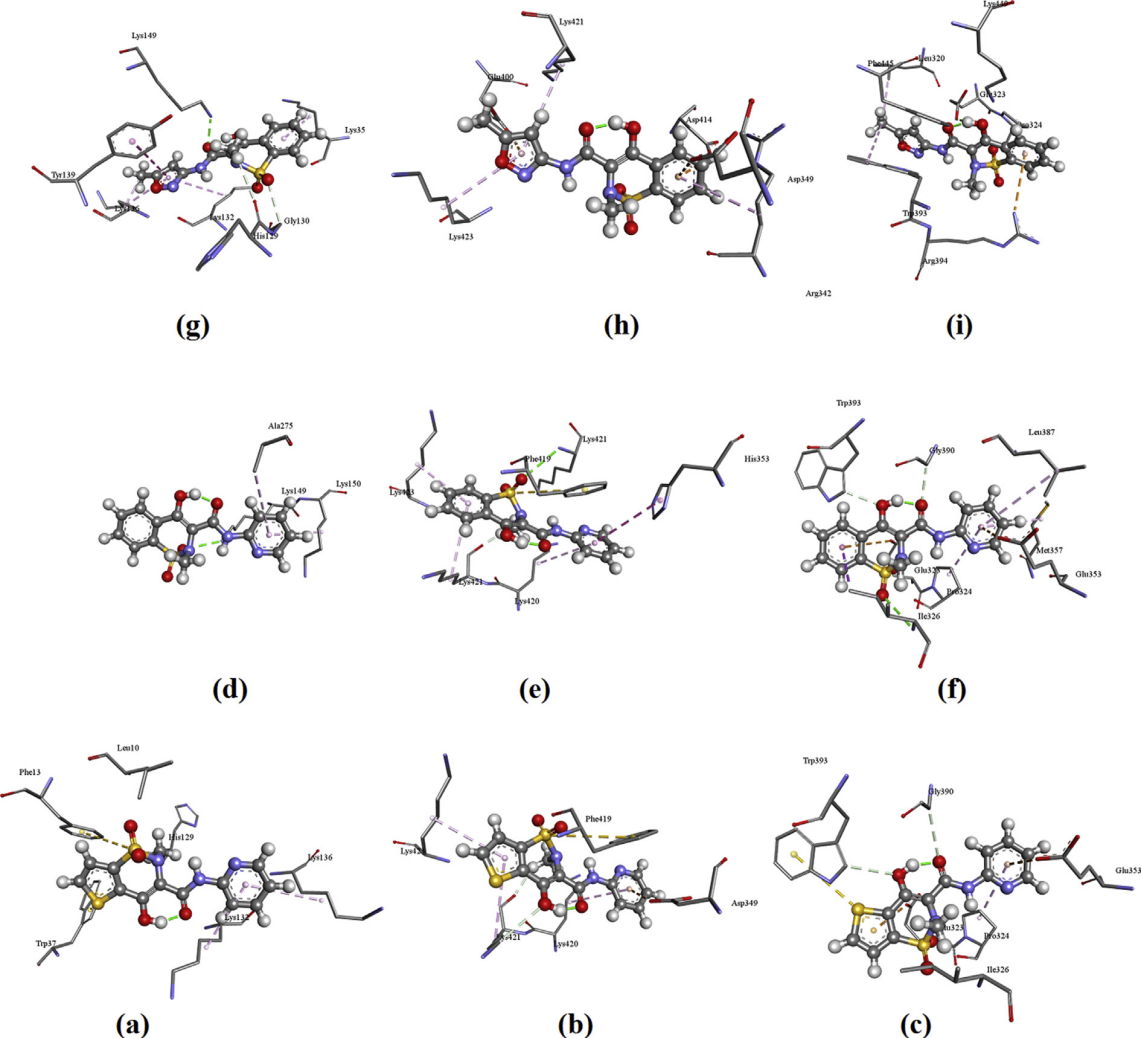
The interactive plot of docked ligands (a) tenoxicam with 3DY9 (b) tenoxicam with 4NZ2 (c) tenoxicam with 2AYR (d) piroxicam with 3DY9 (e) piroxicam with 4NZ2 (f) piroxicam with 2AYR and (g) isoxicam with 3DY9 (h) isoxicam with 4NZ2 (i) isoxicam with 2AYR.

**Fig. 5:**
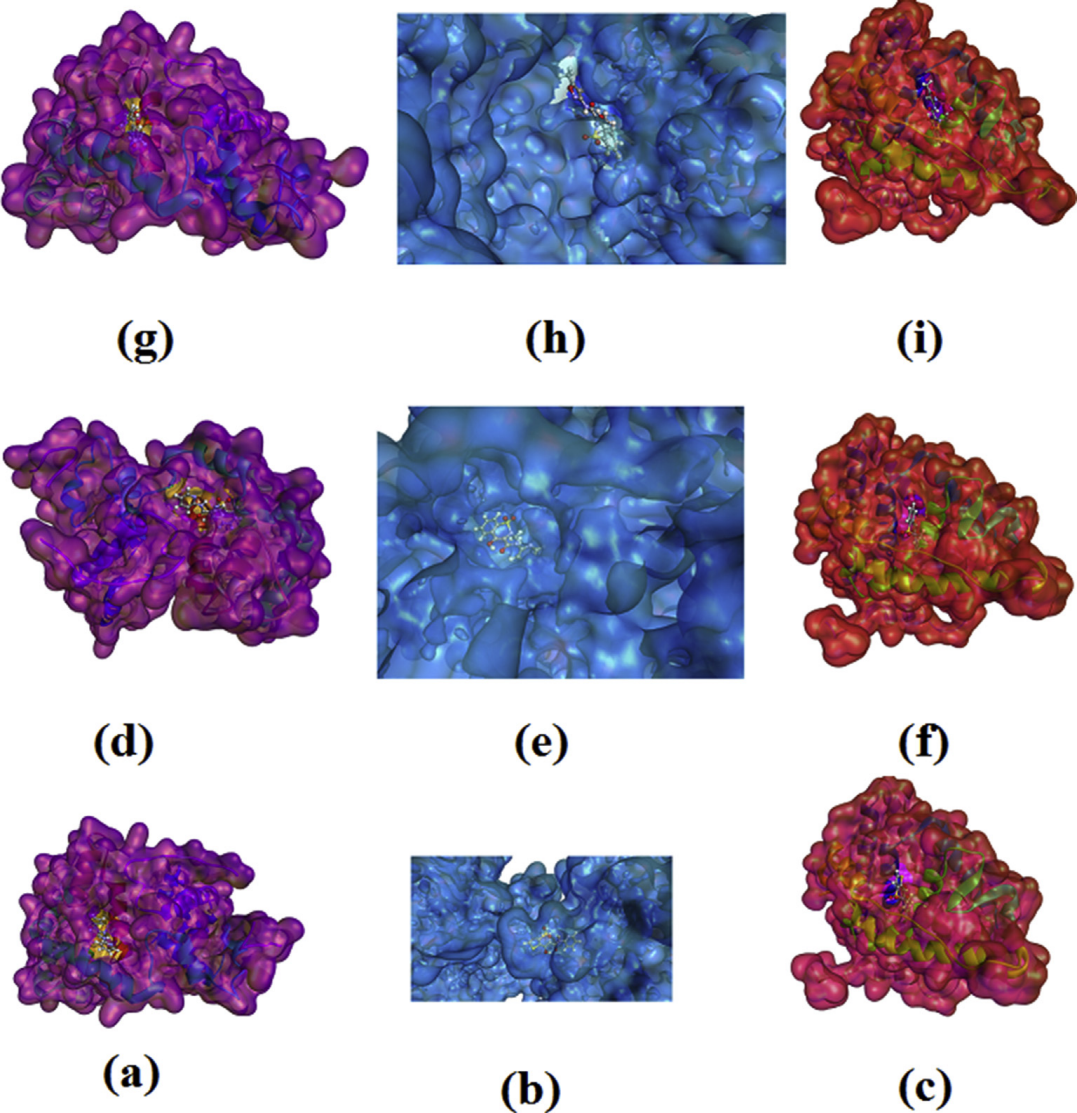
The docked ligands (a) tenoxicam with 3DY9 (b) tenoxicam with 4NZ2 (c) tenoxicam with 2AYR (d) piroxicam with 3DY9 (e) piroxicam with 4NZ2 (f) piroxicam with 2AYR and (g) isoxicam with 3DY9 (h) isoxicam with 4NZ2 (i) isoxicam with 2AYR at the active sites of proteins.

The plot of docked ligand with receptors is shown in Fig. 4 and the docked ligand at the active site of receptors are given in Fig. 5. The docked ligands form a stable complex (Fig. 5) with these receptors with lowest ten minimum conformation of Patch Dock Energy values are tabulated in Table 4. From atomic contact energy value of isoxicam is high in comparison with that tenoxicam and piroxicam and hence isoxicam forms more stable complex with 3DY9, 4NZ2 and 2AYR. For tenoxicam, isoxicam and piroxicam atomic contact energy is high for the protein 2AYR and has high affinity in comparison with other two proteins. The results show that the molecules have inhibitory activity against these receptors.

**Table 4:** The top ten conformation of the complex candidate of ligands.

No.	Global	Attractive	Repulsive	Atomic Contact
	Energy	Vdw	Vdw	Energy
**4.1: Tenoxicam with 3DY9**				
1	-24.54	-10.31	4.74	-10.59
2	-23.13	-9.64	3.26	-9.97
3	-20.96	-8.23	3.68	-9.86
4	-19.95	-8.81	3.95	-8.85
5	-19.00	-9.76	4.36	-7.27
6	-18.42	-8.52	3.30	-6.46
7	-18.03	-10.12	4.77	-6.69
8	-17.80	-8.15	1.05	-5.16
9	-17.65	-6.41	1.11	-6.73
10	-16.05	-11.04	6.24	-4.37
**4.2: Tenoxicam with 4NZ2**				
1	-34.66	-16.32	12.43	-13.55
2	-31.94	-17.60	9.76	-8.63
3	-31.87	-15.12	3.04	-81.0
4	-31.68	-14.01	4.05	-10.74
5	-31.34	-15.29	7.15	-10.20
6	-31.01	-13.51	2.10	-8.87
7	-30.53	-13.98	9.73	-12.44
8	-29.49	-15.72	4.58	-6.27
9	-29.32	-12.55	2.09	-9.18
10	-28.73	-18.44	5.89	-6.08
**4.3: Tenoxicam with 2AYR**				
1	-41.31	-17.01	4.57	-14.33
2	-40.33	-15.82	3.69	-12.34
3	-37.62	-15.70	5.96	-12.74
4	-37.42	-15.77	4.36	-13.93
5	-36.79	-14.47	4.89	-12.86
6	-36.31	-14.68	7.37	-14.24
7	-35.81	-16.05	6.72	-12.25
8	-33.94	-13.08	3.54	-11.40
9	-33.89	-14.92	2.07	-10.81
10	-33.11	-16.88	7.67	-11.98
**4.4: Piroxicam with 3DY9**				
1	-21.21	-9.13	1.14	-6.21
2	-19.67	-9.40	1.72	-6.40
3	-19.37	-8.70	2.61	-8.16
4	-19.32	-9.50	1.49	-5.22
5	-19.26	-9.60	4.33	-7.93
6	-18.28	-8.85	4.22	-7.68
7	-16.16	-6.83	2.99	-6.25
8	-16.08	-11.30	3.95	-3.14
9	-15.74	-11.86	10.39	-5.40
10	-14.54	-7.95	3.79	-6.54
**4.5: Piroxicam with 4NZ2**				
1	-31.03	-15.28	3.95	-7.96
2	-30.98	-12.00	1.99	-9.53
3	-30.62	-15.97	2.28	-6.32
4	-30.06	-19.11	4.45	-5.50
5	-30.05	-16.21	8.99	-10.29
6	-29.95	-16.89	4.80	-5.55
7	-29.60	-13.91	2.61	-8.22
8	-29.33	-14.44	5.50	-9.38
9	-28.70	-16.15	3.84	-5.78
10	-28.66	-15.18	4.96	-7.65
**4.6: Piroxicam with 2AYR**				
1	-37.85	-17.33	5.90	-12.41
2	-36.08	-16.48	4.57	-10.83
3	-35.96	-17.16	4.34	-11.19
4	-34.04	-14.50	4.95	-11.94
5	-33.99	-15.73	5.94	-12.53
6	-33.99	-15.11	6.47	-11.55
7	-33.51	-3.65	3.54	-11.09
8	-33.12	-15.10	2.21	-7.97
9	-31.94	-14.23	1.24	-7.53
10	-31.02	-12.45	1.87	-10.00
**4.7: Isoxicam with 3DY9**				
1	-26.00	-11.53	3.63	-10.59
2	-25.72	-13.30	3.88	-8.13
3	-23.43	-8.48	1.41	-8.64
4	-19.41	-11.68	5.27	-5.59
5	-19.35	-9.10	2.36	-7.89
6	-19.18	-10.49	3.90	-6.91
7	-19.07	-8.18	3.38	-7.26
8	-18.81	-8.40	4.97	-8.39
9	-18.60	-12.28	7.80	-5.68
10	-18.36	-7.93	0.54	-7.12
**4.8: Isoxicam with 4NZ2**				
1	-37.12	-16.16	2.35	-10.90
2	-34.72	-13.37	2.18	-12.16
3	-33.12	-12.59	0.90	-9.56
4	-32.08	-15.24	5.98	-9.06
5	-31.53	-15.55	4.47	-8.39
6	-29.81	-16.07	2.44	-5.55
7	-29.54	-13.01	0.78	-8.32
8	-28.60	-16.09	8.45	-8.67
9	-27.16	-14.20	5.24	-8.46
10	-27.05	-12.26	2.32	-8.50
**4.9: Isoxicam with 2AYR**				
1	-42.31	-19.70	5.02	-12.99
2	-38.46	-16.62	2.34	-9.76
3	-34.63	-14.96	2.06	-9.48
4	-34.52	-15.48	3.75	-10.61
5	-33.93	-14.41	1.86	-9.98
6	-33.62	-15.27	3.42	-11.22
7	-30.98	-14.96	7.86	-9.62
8	-30.94	-12.87	5.08	-12.59
9	-29.64	-11.61	3.41	-10.35
10	-29.61	-15.41	4.42	-9.28

## 4 Conclusion

The spectroscopic analysis of tenoxicam, piroxicam and isoxicam are reported. The theoretical normal modes of vibrations based on DFT theory has been investigated and compared with the experimental values. NBO analysis was carried out on the molecule to find the interactions and found that these compounds are highly stable due to hyperconjugative interactions. Simulated electronic spectra show that there is an intense peak in red shift region due to pi to anti-bonding π-electron transition and a weak peak due to lone pair to anti-bonding orbital transition. The lower value of HOMO and LUMO energy gap describes the stability and biological activity of the tenoxicam, piroxicam and isoxicam compounds. Reactive sites were obtained from MEP, which indicates that there are enough sites for nucleophilic and electrophilic interaction in the molecules, which is very important to show biological activities. Finally the molecular docking shows the ligands have good pharmacological properties with the proteins. From atomic contact energy and global energy values more stable complex are identified.

## Declarations

### Author contribution statement

Y. Shyma Mary, Y. Sheena Mary, K.S. Resmi, Renjith Thomas: Conceived and designed the analysis; Analyzed and interpreted the data; Wrote the paper.

### Funding statement

This research did not receive any specific grant from funding agencies in the public, commercial, or not-for-profit sectors.

### Competing interest statement

The authors declare no conflict of interest.

### Additional information

No additional information is available for this paper.
